# Associations of plasma persistent organic pollutants with brain atrophy, cognitive decline and risk of dementia in older adults

**DOI:** 10.1002/alz70860_106422

**Published:** 2025-12-23

**Authors:** Sophie Lefèvre‐Arbogast, Pierre Scala, Aurore Zorilla, Catherine Helmer, Jade Chaker, Arthur David, Fabien Mercier, Cécilia Samieri

**Affiliations:** ^1^ Bordeaux Population Health Research Center, Inserm U1219, University of Bordeaux, Bordeaux, NA, France; ^2^ Univ. Bordeaux, Inserm, Bordeaux Population Health Research Center, UMR 1219, Bordeaux, France; ^3^ Univ. Rennes, Inserm, EHESP, Irset (Institut de Recherche en Santé, Environnement et Travail) ‐ UMR_S 1085, Rennes, NA, France; ^4^ Bordeaux Population health research center (BPH) INSERM U1219, Bordeaux, NA, France; ^5^ Bordeaux Population health research center (BPH) INSERM U1219, Bordeaux, France; ^6^ Univ. Bordeaux, Inserm, BPH, U1219, Bordeaux, France; ^7^ Univ Rennes, Inserm, EHESP, Irset (Institut de Recherche en Santé, Environnement et Travail), Rennes, NA, France

## Abstract

**Background:**

Persistent Organic Pollutants (POP), including polychlorinated biphenyls (PCB) and organochlorine pesticides (OCP), are established neurotoxicants, especially during developmental periods. Mechanisms of actions include disruption in neurotransmitter systems, calcium dyshomeostasis, oxidative stress and certain OCPs also promote amyloidogenesis in experimental models. Yet, it remains uncertain whether low‐grade environmental exposures in the general population increase the risk to develop brain aging pathologies. We assessed the prospective associations of plasma POP concentrations with three dementia‐related outcomes in a population‐based cohort of older adults.

**Method:**

We included 515 participants from the French Three‐City Study, free of dementia at baseline at the time of blood measurements (1999‐2000, mean age 72.5; 19% APOE‐ɛ4 carriers), who underwent up to 8 repeated assessments of cognitive function and dementia over 17 years and 3 neuroimaging examinations over 10 years. Plasma concentrations of 12 OCPs and 15 PCBs were measured by GC/MS/MS, and a POP score summarizing overall exposure was derived via factorial analysis. Associations with dementia risk, and longitudinal changes in cognitive function (composite measure of four tests) and volume of the medial temporal lobe (MTL) were analyzed using Cox and linear mixed models, adjusted for baseline age, sex, education, APOE genotype, body mass index and total lipid concentrations.

**Result:**

Median plasma concentrations were in accordance with expected values in the population age range. In multivariable‐adjusted models, neither individual POPs nor the POP factorial score were significantly associated with any dementia‐related outcome. Significant interactions were observed between APOE genotype and highly‐chlorinated PCBs (congeners 180, 194, and 196‐203) across all outcomes (*p* for interaction ≤0.05): adverse associations were seen in carriers of the APOE‐ɛ4 allele (e.g. for each SD increase in PCB 194, HR [95% CI] for dementia = 1.51 [1.03‐2.21]; β coefficient [95% CI] for cognitive decline and MTL atrophy = ‐0.15 [‐0.34; 0.04] and ‐0.42 [‐0.74; ‐0.10] SD per decade, respectively), whereas opposing trends were observed in non‐carriers.

**Conclusion:**

Overall, this prospective study does not provide robust evidence to support an adverse association between low‐grade environmental exposure to POPs in the general population and the risk of all‐cause dementia, cognitive decline, or brain atrophy in older adults.

